# A Manual of Therapeutics

**Published:** 1886-10

**Authors:** 


A MANUAL OF THERAPEUTICS. *Considered with Reference to Articles of the Materia Medica. By* EDWARD JOHN WARING, C. I. E., M. D., *Fellow of the Royal College of Physicians, London. Edited by* DUDLEY W. BUXTON, M. D., B. S. (*Edin*). *Philadelphia:* P. BLAKISTON, SON & CO. *Chicago:* W. T. KEENER. *Fourth edition. Small octavo, pp. xxix, 666. 1886.*

In too many cases we pick up a so-called "new edition" of an old work and find it merely a reprint; this is not, however, the case with the present one. It has been thoroughly revised, and much new matter added. The searcher will find almost all the latest therapeutic novelties that have stood in any way the test of experience. The references to the literature of the subject are very full and, with the well arranged index, add much to the value of the work.

